# The Influence of Reducing Disease Activity Score on Cervical Spine Deformity in Rheumatoid Arthritis: A Systematic Review

**DOI:** 10.1155/2022/9403883

**Published:** 2022-04-15

**Authors:** Anna B. Veldman, Cornelia F. Allaart, Carmen L. A. Vleggeert-Lankamp

**Affiliations:** ^1^Department of Neurosurgery, Leiden University Medical Centre, Leiden, Netherlands; ^2^Department of Rheumatology, Leiden University Medical Centre, Leiden, Netherlands; ^3^Department of Neurosurgery, The Hague Medical Centre and HAGA Teaching Hospital, The Hague, Netherlands; ^4^Department of Neurosurgery, Spaarne Hospital Haarlem/Hoofddorp, Netherlands

## Abstract

**Background:**

Rheumatoid arthritis (RA) can cause deformity in particularly the craniocervical but also in the lower cervical region.

**Objectives:**

The aim of this study is to give an overview of current literature on the association of disease activity score (DAS) and the prevalence and progression of rheumatoid arthritis-associated cervical spine deformities.

**Methods:**

A literature search was done in PubMed, Embase, and Web of Science using a sensitive search string combination (Supplemental File). Studies describing the association between DAS and the incidence and progression of atlantoaxial subluxation, vertical subluxation, and subaxial subluxation were selected by predefined selection criteria, and risk of bias was assessed using a Cochrane checklist adjusted for this purpose.

**Results:**

Twelve articles were retrieved, and risk of bias on study level was low to moderate. In the eight longitudinal studies, patients demonstrated high DAS at baseline, which decreased upon treatment with medication: cervical deformity at the end of follow-up was associated with higher DAS values. The four cross-sectional studies did not demonstrate a straightforward correlation between DAS and cervical deformity. Deformity progression was evaluated in three studies, but no convincing association with DAS was established.

**Conclusion:**

A positive association between prevalence of cervical spine deformities and high disease activity was demonstrated, but quality of evidence was low. Progression of cervical deformity in association with DAS control over time is only scarcely studied, and future investigations should focus on halting of deformity progression.

## 1. Introduction

Rheumatoid arthritis (RA) is known for its destructive influence on the cervical spine anatomy [1]. Inflammation of synovial tissue and release of inflammatory cytokines can result in laxity of the ligaments, progressive joint destruction, and erosion of the bone [2]. As a result, abnormal mobility can develop into atlantoaxial subluxation (AAS) and/or vertical subluxation (VS) in the upper cervical spine and to subaxial subluxation (SAS) at the lower cervical levels [3]. This may cause medullary compression, which can lead to sensory and motor dysfunction, disability of arms and legs, spasms, and pain.

In current rheumatology care, a decrease in rheumatoid arthritis-related peripheral joint deformities is observed. This is ascribed to improvements in treatment aimed at achieving low disease activity, in particular with the biological disease-modifying antirheumatic drugs (bDMARDs), which enable the control of systemic inflammatory processes in RA patients more effectively [3]. In the current treatment policies, DMARDs are not only prescribed to more patients, but also in an earlier stage of the disease, which leads to a more effective decrease in systemic inflammation, represented by disease activity scores (DAS) [4].

In evaluating radiographic structural lesions at the hands and feet of RA patients during the course of the disease, a clear association between a decrease in systemic disease activity parameters and stabilization of joint erosions has been established [5–9]. It is likely that efforts to suppress inflammation in RA in an earlier stage, and more effectively than in previous decades, result not only in less damage to peripheral joints, but also in less damage to the cervical spine. Clinical practice seems to reflect this hypothesis: in contemporary orthopedic and neurosurgical clinics, a decrease in incidence of rheumatoid arthritis-related cervical deformities is observed. Several papers have demonstrated an association of a (decrease in) disease activity, usually being influenced by synthetic or biological DMARDs, and the incidence of cervical spine deformity [6, 10].

However, it remains unclear whether deformity can stabilize, or even reverse, if DAS values are lowered to satisfactory levels. The aim of this study is to systematically review current literature on the association of the course of disease activity scores (DAS) and the prevalence and progression of rheumatoid arthritis-associated cervical spine deformities.

## 2. Material and Methods

The systematic review was conducted in accordance with the Preferred reporting items for systematic reviews and meta-analyses: the PRISMA statement [11].

### 2.1. Search Strategy and Study Selection

In December 2020, the databases PubMed, Cochrane, Embase, Web of Science, and Central were searched for peer-reviewed articles, excluding meeting abstract references, using the search strategy in appendix A based on the following PICO: P, patients suffering from rheumatoid arthritis; I, patients with an increased DAS or DAS28 or DAS44 score; C, patients with a low DAS or DAS28 or DAS44 score or being in remission; and O, cervical deformity, represented by AAS (or AAI), SAS, or VT. Two of the authors (AV and CVL) separately screened the articles by title and abstract, to select studies that met the predefined selection criteria.

Any discrepancy in selection between the two reviewers was resolved in open discussion. The obtained articles were checked for citations of articles missed in the search, so no relevant articles were missed.

Inclusion criteria:

- The article was published in English or Dutch
- The study included patients diagnosed with rheumatoid arthritis (ANCA, TNF, or rheumatoid factor positive)
- The study included the measuring of disease activity in all of the patients
- The study concerned cervical anatomy/deformity diagnosed on cervical fluoroscopy or MRI
- The study was a case control study, cohort study, or randomized controlled trial

Exclusion criteria:

- The study included less than 10 patients
- Meta-analysis or systematic review
- The study had a follow-up period of less than 6 months

### 2.2. Assessment of Quality

The methodological quality of these studies was assessed by two independent reviewers (AV and CVL), using a modified version of the checklist for cohort studies of the Dutch Cochrane Center.

The items reviewed in the assessment, focusing on study level, were definition of patient group (containing information on age, gender, and diagnosis of rheumatoid arthritis), selection bias, allocation bias, and attrition bias (loss to follow-up below 20%). For each item, one point could be attributed, and thus, a maximum score of four points could be achieved by each article.

## 3. Data Extraction

Data from the studies were extracted by two independent reviewers (ABV and CVL) concerning study design, sample size, patient characteristics, disease duration, severity of RA, follow-up, and type of radiological evaluation. Disease activity in a composite score was based on evaluation of 44 or 28 peripheral joints, by evaluating erythrocyte sedimentation rate (ESR) or C-reactive protein (CRP) and by evaluating general health assessment on a visual analogue scale (VAS). Thus, disease activity can be represented as DAS, DAS28-ESR, or DAS28-CRP [12]. The cut-off values differ for DAS and DAS 28; for DAS, activity can be interpreted as low (DAS ≤ 2.4) or high (DAS > 3.7). A DAS < 1.6 corresponds with being in remission. For DAS28, activity can be interpreted as low (DAS28 ≤ 3.2) or high (DAS28 > 5.1). A DAS28 < 2.6 corresponds with being in remission. There is no difference in cut-off values for DAS28 whether it is calculated using ESR or CRP [12].

DAS were evaluated at baseline and during follow up. Radiological scoring (cervical deformity) evaluated the presence and progression of atlantoaxial subluxation (AAS; or sometimes indicated as AAI: atlantoaxial instability), vertical subluxation (VS), and subaxial subluxation (SAS).

Finally, the assessed correlations between DAS and cervical deformity presence and progression, as indicated by the authors, were extracted from the selected articles.

These data were gathered on piloted forms and compared. Any discrepancies were discussed.

## 4. Level of Evidence

The quality of evidence for all outcome parameters were planned to be evaluated using the GRADE (Grading of Recommendations Assessment, Development and Evaluation) approach (according to Atkins et al. [13] and adapted from Furlan et al. [14].

## 5. Results

### 5.1. Search Results

**Figure 1:**
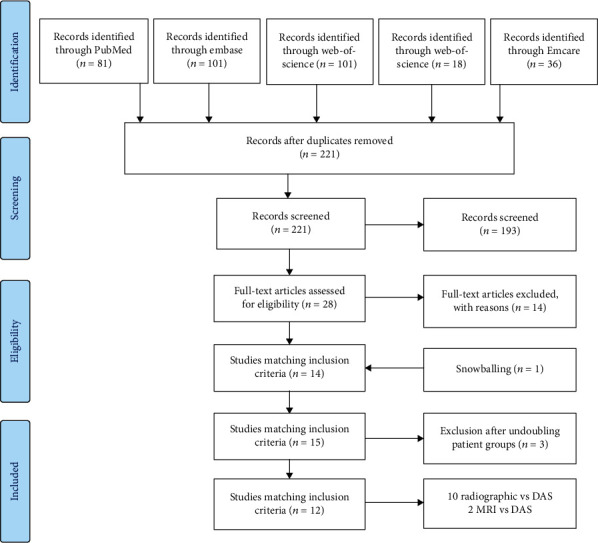
Flow chart applying PRISMA criteria to inclusion of articles.

In the search, 221 articles were identified after duplicates were removed. Titles and abstracts were screened resulting in 28 articles eligible for inclusion. Full-text reading excluded another 14 articles, resulting in the inclusion of 14 articles (Figure 1). In one of these articles, the authors referred to an article that fulfilled the inclusion criteria but was not identified in the search (“snowballing”). This article was added, leading to 15 articles being included. However, amongst these, 4 articles were produced by the same author group [10, 15–17], describing the same correlations in a growing group of patients over the years (2012: 38 patients; 2013: 91 patients; and 2017 and 2019: 151 patients, same population). Therefore, only the 2019 paper is considered in this review.

Consequently, 12 articles are considered in the current review: (a) 7 articles longitudinally describing the correlation between cervical deformity on cervical spine radiographs and disease activity [6, 16, 18–22], including 2 articles describing the same population at 2-year [18] and 5-year follow-up [19], with focus on different aspects of the DAS-cervical deformity association; (b) 2 articles cross-sectionally describing the correlation between cervical deformity on cervical spine radiographs and DAS28 [7, 23] and 1 article describing the correlation of DAS28 measured at baseline and cervical deformity on cervical spine radiographs after years of follow up [24]; and (c) 2 articles describing the correlation between presence of atlantoaxial synovitis on MRI and DAS either longitudinally [25] or in a cross-sectional manner [26].

The number of patients studied varied from 20 to 220, the mean disease duration at baseline varied from 6 months to 11 years, RA severity at baseline varied from “early onset” to an advanced Steinbrocker stage, and the follow-up period varied from 1 to 12 years (Table 1). Most studies evaluated the DAS28 either with ESR or CRP data, and only two studies used the DAS [25, 26].

### 5.2. Risk of Bias

In all studies, the patient group was defined properly, reporting age, gender, duration of disease at baseline, and reporting that the diagnosis RA was according to the American College of Rheumatology criteria [27]. Selection bias was absent in the study of Neva and Kauppi since the patients were randomized [18, 19]. The two studies describing MRI results indicated that they included “consecutive” patients with strict criteria [25, 26].

Allocation bias was absent in the study of Neva et al. [18, 19] and in the studies of Kanayama, Sandstrom, Zoli, and Carotti [6, 22, 25, 26] since the patients were subjected to a strict medication regimen for all included patients. Attrition bias was consequentially not present in retrospective and cross-sectional studies (Table 2). In some studies, evaluation of radiographic images was done by an independent reviewer [18–20, 24].

### 5.3. Definitions of Cervical Deformity

Cervical spine deformity is described with a variety of parameters throughout the articles, but all articles used the parameter atlantoaxial subluxation (AAS). The parameters vertical subluxation (VS) (or atlantoaxial impaction, AAI) and subaxial subluxation (SAS) were also frequently reported and evaluated in this article. Definitions of abnormality differ slightly between studies (Table 3). AAS, measured as the distance from the middle of the posterior border of the anterior part of the C1 arch until the anterior cortex of the odontoid peg (ADI), was considered abnormal if the difference in neutral position exceeded 3 mm [16, 21, 23, 26] or exceeded 3 mm difference in flexion radiographs [6, 7, 18, 19, 22, 24].

VS was considered to be present if the odontoid peg entered more than 0 [20, 23] or 4-5 mm through the foramen magnum [26]; if the Sakaguchi-Kauppi value was grades II, III, or IV [6, 18, 19, 22, 24]; or if the Ranawat value was under 13 mm [7, 16, 21]. SAS was defined as the dislocation of two vertebra in the neutral position of the cervical spine exceeding 2 [7, 16] or 3 mm [18, 19, 22, 24].

Progression of AAS was defined as an increase of the ADI of more than 1 [6] or 2 mm [16, 21], progression of VS was defined as an increase of the Ranawat of more than 0 [6] or 2mm [16, 21], and the progression of SAS was defined as an increase of more than 2 mm [16].

### 5.4. Longitudinal Evaluation of Cervical Deformity and DAS Values

In order to evaluate whether active inflammation, represented by the DAS or DAS28, had an influence on cervical deformity, the seven articles describing the longitudinal correlation between cervical deformity and disease activity are the most informative. In four of those studies, patient groups with recent onset RA are described of which can be assumed that cervical deformity is absent at baseline. No radiographic detectable cervical deformity was evaluated and described by Blom et al. [20] and Sandstrom et al. [22] and assumed in the patient groups described by Neva et al. [18, 19]. With a varying follow up from 2 to 12 years, AAS developed in 2.4 to 8.1% of patients with the DAS ranging between 2.0 and 3.6 (Table 4).

#### 5.4.1. Longitudinal Correlations between Cervical Deformity and DAS in Recent Onset RA

**Figure 2:**
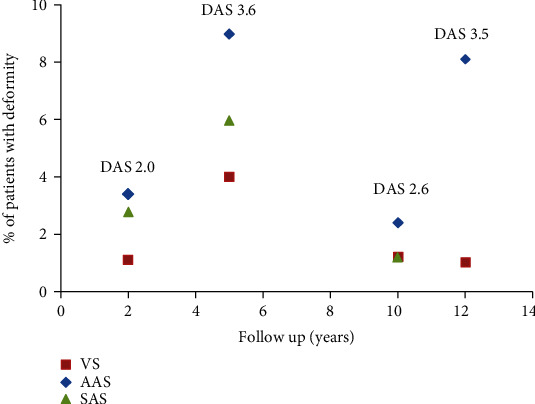
Overview of the correlation between duration of follow up, DAS and % of patients with cervical deformity at the end of follow up in the four articles describing longitudinal follow-up in patient groups with recent onset RA. Red squares represent VS, blue diamonds represent AAS, and green triangles represent SAS.

The patient groups with the highest percentages of AAS, VS, and SAS at the end of follow up had the highest DAS (Figure 2). Neva executed a treatment strategy aiming at lowering systemic inflammation but failed to achieve DAS28-remission in the group of patients that developed cervical deformity during the two-year follow-up period, in contrast to the group without cervical deformity [18]. Kauppi demonstrated that the area under the curve for DAS was significantly higher in the groups that developed AAS, VS, or SAS [19]. Blom reasoned that there were so many missing values in their database that longitudinal follow-up was not valuable; they could only conclude that in patients without AAS or VS at the nine-year follow-up timepoint, the mean DAS28 at the three-year follow-up timepoint was lower [20]. They however failed to demonstrate this at the twelve-year follow-up timepoint. Sandstrom concluded that AAS, VS, and SAS occurrence was so low in their patient group, even after 10-year follow-up, that no meaningful correlations to DAS28 could be made [22].

#### 5.4.2. Longitudinal Correlations between Cervical Deformity and DAS Values in Advanced Stage of RA

The other three articles longitudinally describing cervical deformity and DAS over time demonstrate the same pattern. They reported on patient groups that had been suffering from RA for 10 [16], 11 [6], and 17 [21] years. In these populations, 33 to 50% of patients did not demonstrate any deformity at that timepoint, and 6-8% of these patients developed AAS during 3- to 4.5-year follow-up. Horita described that 24% of the patients that already had cervical deformity demonstrated progression of deformity during the 3-year follow-up and that the DAS of those patients was significantly higher (3.2, range 1.1–4.0) than the DAS of patients who did not demonstrate progression of deformity (2.1, range 1.1–3.8; *p* < 0.001) [21]. Kaito described that in the 50% of patients with cervical deformity, 81-86% of patients demonstrated progression although the mean DAS28 at final follow up was 2.6 (representing clinical remission). It was not reported whether the values differed in patients with or without progression [16]. Kanayama reported that 34% of patients with AAS on baseline progressed in AAS and that 21% of patients with VS progressed in Ranawat score and that the DAS28 was higher in patients who demonstrated progression of cervical deformity (mean 4.2 ± 1.1 vs 3.1 ± 1.3), though seemingly not significant [6].

### 5.5. Cross-Sectional Evaluation of Cervical Deformity and DAS Values

The cross-sectional papers report on populations suffering from RA for 10 [23], 11 [7], and 13 [24] years. They demonstrate a prevalence of AAS of 10 to 36%, of VS of 5 to 10%, and of SAS of 5 to 13%. The correlation with DAS is not straightforward: Takahashi reports low DAS values [7] and Neva reports moderate DAS values [24] without a difference between patients with and without deformity. Takahashi concluded that suffering from RA for over ten years was a risk factor for developing cervical deformity, while Neva denies that duration of RA correlates to the development of AAS.

Younes evaluated deformity on cervical radiographs and on MRI and reported the presence of synovitis in the upper cervical segments, while the radiographs did not demonstrate deformity (yet). Adding the numbers of patients with synovitis and patients with cervical deformity on radiograph, they state that 36% of patients have AAS. The mean DAS28 in this study population was 4.79 ± 1.62, without a significant difference in the percentage of patients with a DAS higher than 3.2 in the patients with AAS (78%) compared to the patients without AAS (86%) [23].

### 5.6. Correlations between Atlantoaxial Synovitis on MRI and DAS Values

Finally, in the articles that evaluated MRI of the cervical spine of RA patients, active synovitis was reported in 25% of patients with recent onset RA [25, 26]; additionally, performed radiography of the cervical spine did not demonstrate cervical deformity [26] (Table 4). The mean DAS was high in all patients, although it was reported that in patients with deformity, the DAS was significantly higher than in patients without deformity. Zoli reported additionally that after starting medication, aiming at lowering systemic inflammation, one patient demonstrated complete and one patient partial regression of synovial involvement [25].

### 5.7. Correlation of Cervical Deformity and Peripheral Joint Deformity

Four of the ten articles that studied RA deformity on radiographs of the cervical spine demonstrate a positive correlation between cervical and peripheral joint deformity [7, 18, 20, 24]. Only Younes fails to demonstrate such a correlation in a patient group suffering from RA for circa ten years [23]. Neva states that in the patient group that has been suffering from RA for five years, the Larsen score is predictive for the development of AAS [24].

In the two articles that compared cervical deformity on MRI with DAS, it was demonstrated that cervical synovitis correlated to erosions in the joints of the hands and feet [25, 26].

## 6. Discussion

Careful evaluation of literature does not provide us with a satisfactory answer to the question whether control of systemic disease activity in rheumatoid arthritis can prevent progression of RA associated cervical spine deformity. The overall picture however suggests that disease activity, represented by DAS or DAS28, in RA patients with cervical deformity was higher than in those without deformities, although the reported differences were small.

A limitation to the conclusions that could be drawn from this systematic review is that the baseline cervical deformity was not consequently described. Only two studies evaluated the association of DAS in the early stage of disease and cervical deformity after 10-12 years follow-up [20, 24], but due to the abundance of missing values, these studies failed to demonstrate a convincing positive correlation. In future studies, it is advisable to correlate disease activity over time with deformity at the end of follow up. This can be done by using the AUC of DAS values over time. Two studies reported on an AUC value of DAS [19, 24], but again, conflicting results were reported. Kauppi showed a higher DAS-AUC in patients with deformity [19], while Neva could not appoint a positive correlation between the DAS-AUC in the first years of RA with cervical deformity at the end of follow up. Again, a study is set up in which data in individual patients between DAS and deformity can strengthen conclusions.

Another limitation is the scarcity of literature on this topic and the variance in set-up of the available studies. Two studies evaluated patients that already developed deformity; Kanayama reported a higher DAS in patients in which deformity progressed (at least 1 mm increase in ADI or Ranawat after one-year follow-up) in comparison to patients in which AAS and VS remained the same (less than 1 mm increase) [6]. Kaito reports the opposite: halting of progression of deformity could not be achieved; almost 80% of patients with deformity demonstrated progression in deformity though systemic inflammation was tempered [16]. A firm conclusion cannot be drawn, particularly because follow-up was short, and both the differences in AAS and VS and those between DAS in the progressive and nonprogressive group were very small.

The question that remains is whether deformity, once it has developed, can be halted by suppressing disease activity, possibly even to remission of disease. A barrier in studying this hypothesis is that with the current successful treatment-to-target regimes [28], the percentage of patients that develop deformity is low, as demonstrated in this review [18–20, 22]. Therefore, in future studies on this topic, large groups of patients have to be included, in order to include enough patients in which treatment-to-target therapy fails and in which patients consequently have high DAS. Moreover, future studies should monitor DAS over many timepoints in order to get a good overview of the decrease in DAS, remission, and flares. This should be combined with radiographs at baseline, at intervals, and at the end of sufficiently long follow-up periods.

The paucity of available studies prevented us from performing a meaningful meta-analysis of the included studies. This is caused by the low quality of evidence, as well as the different approaches of diagnosing cervical spine deformity and measuring systemic disease activity in current literature on this topic.

The DAS is not the only parameter that is an indicator for systemic inflammation. MMP3 has also been evaluated in several of the articles studied in this review. Kanayama even demonstrated that the decline in MMP3 was more impressive than the decline in DAS and that it demonstrated a clearer difference between patients with and without progression of cervical deformity [6]. Kauppi performed a multiple regression in a group with recent onset RA and evaluated the correlation of cervical deformity to other parameters and reported that a worse score on HAQ at baseline was predictive for deformity after 5 years follow-up with an OR of 5.81 (1.64-20.52) [19]. The limitation of this study was, however, that no radiographs of the cervical spine were obtained at baseline. It might thus be that the HAQ was worse in those patients that already suffered from cervical synovitis, or even deformity, at baseline. This indicates that in future studies, cervical deformity should not only be correlated to DAS as systemic parameter, but it would be valuable to also study correlations with MMP3, self-reported disability, treatment strategy, and/or hand-and-foot erosions.

The goal of finding correlations between certain parameters and cervical deformity after follow up in RA patients is that patients in which progression of deformity is very likely can be appointed and that they can be treated more adequately. Medication treatment can be more aggressive, systemic inflammation more intensely monitored, and, in absence of accomplishing a satisfactory low systemic inflammation status, surgery can be offered in a stage in which deformity is still mild. Once the upper cervical spinal elements are fused by instrumentation, RA pannus diminishes, atlantoaxial deformity stops, and possible compression of the neural structures is prevented [29, 30].

Introduction of biologicals in the treatment of RA has achieved impressive improvement in lowering systemic disease. This is being held responsible for the decrease in prevalence of cervical deformity. This is at least partially true: there is a clear correlation between low DAS values and less cervical deformity. The current overview of literature can however not confirm the hypothesis that progression of deformity can be halted by lowering systemic inflammation. Drawing a conclusion is hindered by the poor quality of data to confirm of reject of the hypothesis. Another hypothesis that may (partially) explain the decrease in cervical deformity in RA patients is that the treatment with biologicals has abandoned the intense treatment with glucocorticosteroids, which have been demonstrated to coincide with cervical spine deformity [31].

## 7. Conclusion

Lowering disease activity in patients with rheumatoid arthritis has demonstrated to prevent cervical spine deformity with low-quality evidence, but lowering DAS values could not be demonstrated to halt progression with very low-quality evidence. It is important that the role of DAS in predicting cervical spine deformity development and progression is controversial, and other predictors should be identified in further research. In order to manage expectations on cervical deformity in RA patients optimally, it is crucial that the role of disease activity in cervical spine deformity is further evaluated.

## Figures and Tables

**Table 1:** Prevalence of cervical spine deformity.

	*n*	% *F*	Age (yr) ± SD [range]	Disease duration (yr) [range]	Disease activity score	RA severity (Steinbrocker I:II:III:IV) at baseline	Radiological evaluation	Follow-up (mos) [range]
	Correlation between cervical deformity on cervical spine radiographs and disease activity from baseline to follow-up							
Neva (2000) [18]	176	63	46 ± 10	0.6 [0.2-1.8]	DAS28-ESR #	Early onset	X cerv (at FU)	24
Kauppi (2009) [19]	149	66	48	0.5 [0.3-0.8]	DAS28-ESR #	Early onset	X cerv	60
Kanayama (2010) [6]	47	77	53 ± 13.4	11 ± 10	DAS28-ESR	2 : 9 : 22 : 14	X cerv	12
Blom (2013) [20]	196	64	51.6 ± 13.7	Max 12 Mos	DAS28-ESR	Early onset	X cerv	144
Kaito (2019) [16]	101	83	57 ± 10 [31-75]	10,7 [0.3–42]	DAS28-CRP	5 : 20 : 41 : 35	X cerv	53 [24-96]
Horita 2019 [21]	49	90	59 [30-81]	17.5 [1-46]	DAS28-CRP	0 : 0 : 13 : 36	X cerv	39 [12-69]
Sandstrom (2020) [22]	85	67	48 ± 10	4	DAS28-ESR		X cerv	120
	Correlation between cervical deformity on cervical spine radiographs and DAS-28 in a cross-sectional manner							
Neva (2003) [24]	103	67	45-54	0.5	DAS28-ESR	Early onset	X cerv	96-156 (##)
Younes (2009) [23]	40	78	55.2 ± 11.9	10 ± 7.9	DAS28-CRP		X cerv/MRI cerv	Cross sectional
Takahashi 2014 [7]	220	83	64 [25-84]	11.1 [0.1-57.2]	DAS28-CRP	21 : 26 : 35 : 18	X cerv	Cross sectional
	Correlation between presence of atlantoaxial synovitis on MRI and DAS-28							
Zoli (2011) [25]	20	85	54	0.5	DAS	Early onset	MRI cerv	18
Rotti (2019) [26]	50	74	58.2 [36-79]	0.8 [0.41-1]	DAS	Early onset	MRI cerv	Cross sectional

Overview of patient demographics in the studies. MRI was performed with a 1.5 Tesla machine producing fast spin-echo T1-weighted images with fat suppression, with [25] or without [26] intravenous contrast. The MRI scan allowed assessment of the presence of synovitis and erosive joint damage in the upper cervical region. (#) Calculated with DAS28 calculator using the number of swollen joints, number of tender joints, and ESR, (##) recruited in the database 8 to 13 years before; at that time, they were diagnosed with RA 5.6 to 6.4 months before. na: not applicable.

**Table 2:** Risk of bias in the studies.

Study	Score on risk of bias scale	Well-defined patient group	Absence of selection bias	Absence of allocation bias	Absence of attrition bias
Neva (2000) [18]	++++	+	+	+	+
Kauppi (2009) [19]	+++	+	+	+	-
Kanayama (2010) [6]	++	+	-	+	-
Blom (2013) [20]	++	+	+	-	-
Kaito (2019) [16]	++	+	-	-	+
Horita (2019) [21]	++	+	-	-	+
Sandstrom (2020) [22]	+++	+	-	+	+
Neva (2003) [24]	++	+	-	-	+
Younes (2009) [23]	++	+	-	-	+
Takahashi (2014) [7]	++	+	-	-	+
Zoli (2011) [25]	++++	+	+	+	+
Carotti (2019) [26]	++++	+	+	+	+

**Table 3:** Definitions of cervical deformity.

	Definitions of pathology	Definitions of progression of pathology
AAS	(i) Distance from the middle of the posterior border of the anterior part of the C1 arch until the anterior cortex of the odontoid peg (ADI) exceeding 3 mm in *neutral* position [16, 21, 23, 26] (ii) Distance from the middle of the posterior border of the anterior part of the C1 arch until the anterior cortex of the odontoid peg (ADI) exceeding 3 mm in *flexed* position [6, 7, 18, 19, 22, 24]	(i) Increase of the ADI of more than 1 mm [6] (ii) Increase of the ADI of more than 2 mm [16, 21]
VS	(i) Odontoid peg entering more than 0 [20, 23] mm through the foramen magnum [20, 23] (ii) Odontoid peg entering more than 4-5 mm through the foramen magnum [26] (iii) Sakaguchi-Kauppi value being grades II, III, or IV [6, 18, 19, 22, 24] (iv) Ranawat value being less than 13 mm [7, 16, 21]	(i) Increase of the Ranawat of more than 0 mm [6] (ii) Increase of the Ranawat of more than 2mm [16, 21]
SAS	(i) Dislocation of two vertebra in the neutral position of the cervical spine exceeding 3 mm [7, 16] (ii) Dislocation of two vertebra in the neutral position of the cervical spine exceeding 3 mm [18, 19, 22, 24]	(i) Increase the SAS of more than 2 mm [16]

**Table 4:** Cervical spine deformity and DAS overview.

	*n*	Deformity none		AAS		AAS + VS/AAI		VS/AAI		DAS baseline	DAS final
		Baseline	Progression	Baseline	Progression	Baseline	Progression	Baseline	Progression		
	Correlation between cervical deformity on cervical spine radiographs and DAS28 from baseline to follow-up										
Neva (2000) [18]	176	100% #	AAS 3.4% AAI 1.1% SAS 2.8%							5.97^	2.01^
Kauppi (2009) [19]	149	100% #	AAS 9% AAI 4% SAS 6%							5.53–5.65	In pts - deformity AUC 3.1 In pts + deformity AUC 3.6^∗^
Kanayama (2010) [6]	47			Mean ADI 4.1 ± 2.2	Mean ADI 4.5 ± 2.3∗∗, 34% of patients progression of ADI			Mean Ranawat 13.6 ± 2.6	Mean Ranawat 13.4 ± 2.7∗∗, 21% of patients progression of Ranawat	5.71	Nonprogressive 3.11 ± 1.27, progressive 4.18 ± 1.06
Blom (2013) [20]	196	100% (R1)	3 yrs: AAS 4.8%, AAI 0% 6 yrs: AAS 5.9%, AAI <1% 9 yrs: AAS 7.3%, AAI <1% 12 yrs: AAS 8.1%, AAI <1%							5.45 ± 1.38	Non progressive at 9 yrs: 3.69, progressive at 9 yrs: 3.51
Kaito (2019) [16]	101	50%	None: 92% AAS 8%	32%	None 19% AAS 34% VS 63% SAS 9%	12%	None 17% AAS 8% VS 58% SAS 25%	7%	None 14% AAS 14% VS 71% SAS 14%	4.4 ± 0.8	2.6 ± 0.8
Horita (2019) [21]	49	33%	6% (R2)			67%	24% ∗∗			In pts – progression in deformity 3.1 In pts + progression in deformity 4.1^∗^	In pts – progression in deformity 2.1 In pts + progression in deformity 3.2^∗^
Sandstrom (2020) [22]	85	100%	None: AAS 2.4% AAI 1.2% SAS 1.2%							5.5 ± 0.9 and 5.6 ± 1.4	< 2.6 (R3)
	Correlation between cervical deformity on cervical spine radiographs and DAS28 in a cross-sectional manner										
Neva (2003) [24]	85	100% #	None: 83% AAS 10% AAI 5% SAS 5%							In pts - deformity 3.3 In pts + deformity 3.5	In pts - deformity 3.5 In pts + deformity 4.5
Younes (2009) [23], Xcerv	50			22.5%	Na	SAS: 10%	Na	10%	Na	MRI and Xcerv findings together: 36% AAS In pts with AAS 78% of pts had DAS> 3.2 and in pts without AAS 86% of pts had DAS> 3.2	Na
Younes (2009) [23], MRI				17.5% active synovitis on MRI, 15% fibrous pannus, 30% hypervascular pannus, 20% of patients demonstrated AAS on MRI							
Takahashi (2014) [7]	220			36%	Na	SAS: 13%	Na	10%	Na	2.66 [1.02 – 6.96]	Na
	Correlation between presence of atlantoaxial synovitis on MRI and DAS										
Zoli (2011) [25]	20	75% no active synovitis		Baseline: 25% active synovitis on MRI						In pts – deformity 3.9 ± 0.2, in pts + deformity 5.0 ± 0.8∗	
Carotti (2019) [26]	50			24% active synovitis on MRI (R4)						In pts – Deformity 4.5 ± 0.5, in pts + deformity 5.7 ± 0.4∗	

Cervical deformity and DAS at baseline and at the end of follow up. The number of patients in the different studies is indicated as well. (#) presumed percentage, regarding the early onset of RA, (^) calculated from data in the article, (∗) significant difference between patients with and without deformity, (∗∗) significant difference compared to baseline value; (R1) remark 1: CWK radiograph available: baseline *n* = 60; 3 years: *n* = 66; 6 years: *n* = 180; 9 years: *n* = 134; 12 years: *n* = 78; (R2) remark 2: Calculated from data in discussion “In the present study, the percentage of patients with any cervical instability at baseline (65.3% of 49 patients) increased to 69.4% at final follow up”; (R3) remark 3: derived from the results section: “the four patients with cervical spine deformity were in sustained remission during the whole follow-up time”; (R4) remark 4: contradictory details are given in the article varying from “no obvious radiological lesions were evident” to “AAS was observed in two of the 12 patients with synovitis on MRI,” na: not applicable.

## Data Availability

The data used to support the findings of this study are included within the article.

## Supplementary Materials

Supplementary Materials The search strategy used for this systematic review has been added as a supplemental file.
