# Supplementary material for: The Influence of Reducing Disease Activity Score on Cervical Spine Deformity in Rheumatoid Arthritis: A Systematic Review
Source: Biomed Res Int. 2022 Apr 15;2022:9403883. doi: 10.1155/2022/9403883 (PMC9033349; doi:10.1155/2022/9403883)
Supplement: Supplementary Materials — The search strategy used for this systematic review has been added as a supplemental file. [file 9403883.f1.pdf]

Appendix: Search String

PubMed

http://www.ncbi.nlm.nih.gov/pubmed?otool=leiden

results: 69

(("Arthritis, Rheumatoid"[Mesh:noexp] OR "rheumatoid arthritis"[tw] OR "rheumatoid"[tw] OR "rheumatic"[tw] OR "arthritis"[tw] OR arthriti*[tw] OR "RA"[tiab]) AND ("Cervical Vertebrae"[mesh] OR cervical*[tw] OR "Cervical Vertebrae/abnormalities"[Mesh] OR "SAS"[tw] OR "subaxilar subluxation"[tw] OR "subaxilar subluxations"[tw] OR "subaxial subluxation"[tw] OR "subaxial subluxations"[tw] OR "AAI"[tw] OR "atlanto axial impaction"[tw] OR "atlantoaxial impaction"[tw] OR "AAS"[tw] OR "anterior atlantoaxial subluxation"[tw] OR "anterior atlanto axial subluxation"[tw] OR "anterior atlantoaxial subluxations"[tw] OR "anterior atlanto axial subluxations"[tw] OR "VT"[tw] OR "vertical translocation"[tw] OR "vertical translation"[tw] OR "vertical translocations"[tw] OR "vertical translations"[tw]) AND ("das28"[tw] OR "das 28"[tw] OR "das28 score"[tw] OR "das28 scored"[tw] OR "das28 scores"[tw] OR "das28 value"[tw] OR "das28 values"[tw] OR "das 28 score"[tw] OR "das 28 scores"[tw] OR "das 28 values"[tw] OR "disease activity score 28"[tw] OR "disease activity"[tw] OR "disease activities"[tw] OR "disease active"[tw] OR "diseases activities"[tw] OR "diseases activity"[tw] OR "illness activity"[tw] OR "Severity of Illness Index"[mesh]) AND ("Time"[mesh] OR "time"[tw] OR time*[tw] OR timing*[tw] OR "long term"[tw] OR "longterm"[tw] OR "short term"[tw] OR "shortterm"[tw] OR followup*[tw] OR "follow up"[tw] OR "Follow-Up Studies"[mesh]))

Archivalia

(("Arthritis, Rheumatoid"[Mesh:noexp] OR "rheumatoid arthritis"[tw] OR "rheumatoid"[tw] OR rheumat*[tw] OR "arthritis"[tw] OR arthriti*[tw] OR "RA"[tiab]) AND ((("Cervical Vertebrae"[mesh] OR cervical*[tw]) AND ("deformities"[tw] OR "deformity"[tw] OR "Spinal Curvatures"[Mesh] OR "spinal curvatures"[tw] OR "spinal curvature"[tw] OR "spine curvatures"[tw] OR "spine curvature"[tw])) OR "Cervical Vertebrae/abnormalities"[Mesh] OR "SAS"[tw] OR "subaxilar subluxation"[tw] OR "subaxilar subluxations"[tw] OR "subaxial subluxation"[tw] OR "subaxial subluxations"[tw] OR "AAI"[tw] OR "atlanto axial impaction"[tw] OR "atlantoaxial impaction"[tw] OR "AAS"[tw] OR "anterior atlantoaxial subluxation"[tw] OR "anterior atlanto axial subluxation"[tw] OR "anterior atlantoaxial subluxations"[tw] OR "anterior atlanto axial subluxations"[tw] OR "VT"[tw] OR

"vertical translocation"[tw] OR "vertical translation"[tw] OR "vertical translocations"[tw] OR "vertical translations"[tw]) AND ("das28 score"[tw] OR "das28 scored"[tw] OR "das28 scores"[tw] OR "das28 value"[tw] OR "das28 values"[tw] OR "das 28 score"[tw] OR "das 28 scores"[tw] OR "das 28 values"[tw] OR "disease activity score 28"[tw] OR "disease activity"[tw] OR "disease activities"[tw] OR "disease active"[tw] OR "diseases activities"[tw] OR "diseases activity"[tw] OR "illness activity"[tw]))

(("Arthritis, Rheumatoid"[Mesh:noexp] OR "rheumatoid arthritis"[tw] OR "rheumatoid"[tw] OR rheumat*[tw] OR "arthritis"[tw] OR arthriti*[tw] OR "RA"[tiab]) AND ((("Cervical Vertebrae"[mesh] OR cervical*[tw]) AND ("deformities"[tw] OR "deformity"[tw] OR "Spinal Curvatures"[Mesh] OR "spinal curvatures"[tw] OR "spinal curvature"[tw] OR "spine curvatures"[tw] OR "spine curvature"[tw])) OR "Cervical Vertebrae/abnormalities"[Mesh] OR "SAS"[tw] OR "subaxilar subluxation"[tw] OR "subaxilar subluxations"[tw] OR "subaxial subluxation"[tw] OR "subaxial subluxations"[tw] OR "AAI"[tw] OR "atlanto axial impaction"[tw] OR "atlantoaxial impaction"[tw] OR "AAS"[tw] OR "anterior atlantoaxial subluxation"[tw] OR "anterior atlanto axial subluxation"[tw] OR "anterior atlantoaxial subluxations"[tw] OR "anterior atlanto axial subluxations"[tw] OR "VT"[tw] OR "vertical translocation"[tw] OR "vertical translation"[tw] OR "vertical translocations"[tw] OR "vertical translations"[tw]))

MEDLINE

http://gateway.ovid.com/ovidweb.cgi?T=JS&MODE=ovid&NEWS=n&PAGE=main&D=prmz

Embase

http://ovidsp.ovid.com/ovidweb.cgi?T=JS&PAGE=main&MODE=ovid&D=oemezd

(("Rheumatoid Arthritis"/ OR "rheumatoid arthritis".mp OR "rheumatoid".mp OR "rheumatic".mp OR "arthritis".mp OR arthriti*.mp OR "RA".ti,ab) AND (exp "Cervical Vertebra"/ OR cervical*.mp OR "SAS".mp OR "subaxilar subluxation".mp OR "subaxilar subluxations".mp OR "subaxial subluxation".mp OR "subaxial subluxations".mp OR "AAI".mp OR "atlanto axial impaction".mp OR "atlantoaxial impaction".mp OR "AAS".mp OR "atlantoaxial subluxation"/ OR "anterior atlantoaxial subluxation".mp OR "anterior atlanto axial subluxation".mp OR "anterior atlantoaxial subluxations".mp OR "anterior atlanto axial subluxations".mp OR "VT".mp OR "vertical

translocation".mp OR "vertical translation".mp OR "vertical translocations".mp OR "vertical translations".mp) AND ("DAS28"/ OR "DAS28".mp OR "DAS 28".mp OR "das28 score".mp OR "das28 scored".mp OR "das28 scores".mp OR "das28 value".mp OR "das28 values".mp OR "das 28 score".mp OR "das 28 scores".mp OR "das 28 values".mp OR "disease activity score"/ OR "disease activity score 28".mp OR "disease activity".mp OR (exp "Disease activity"/ AND exp "Scoring System"/) OR "disease activities".mp OR "disease active".mp OR "diseases activities".mp OR "diseases activity".mp OR "illness activity".mp OR "Severity of Illness Index"/) AND (exp "Time"/ OR "time".mp OR time*.mp OR timing*.mp OR "long term".mp OR "longterm".mp OR "short term".mp OR "shortterm".mp OR followup*.mp OR "follow up".mp OR "Follow Up"/))

Web of Science

http://isiknowledge.com/wos

(ti=("Rheumatoid Arthritis" OR "rheumatoid arthritis" OR "rheumatoid" OR "rheumatic" OR "arthritis" OR arthriti* OR "RA") AND ts=("Cervical Vertebra" OR cervical* OR "SAS" OR "subaxilar subluxation" OR "subaxilar subluxations" OR "subaxial subluxation" OR "subaxial subluxations" OR "AAI" OR "atlanto axial impaction" OR "atlantoaxial impaction" OR "AAS" OR "atlantoaxial subluxation" OR "anterior atlantoaxial subluxation" OR "anterior atlanto axial subluxation" OR "anterior atlantoaxial subluxations" OR "anterior atlanto axial subluxations" OR "VT" OR "vertical translocation" OR "vertical translation" OR "vertical translocations" OR "vertical translations") AND ts=("DAS28" OR "DAS28" OR "DAS 28" OR "das28 score" OR "das28 scored" OR "das28 scores" OR "das28 value" OR "das28 values" OR "das 28 score" OR "das 28 scores" OR "das 28 values" OR "disease activity score" OR "disease activity score 28" OR "disease activity" OR ("Disease activity" AND "Scoring System") OR "disease activities" OR "disease active" OR "diseases activities" OR "diseases activity" OR "illness activity" OR "Severity of Illness Index") AND ts=("Time" OR "time" OR time* OR timing* OR "long term" OR "longterm" OR "short term" OR "shortterm" OR followup* OR "follow up" OR "Follow Up")) OR (ts=("Rheumatoid Arthritis" OR "rheumatoid arthritis" OR "rheumatoid" OR "rheumatic" OR "arthritis" OR arthriti* OR "RA") AND ti=("Cervical Vertebra" OR cervical* OR "SAS" OR "subaxilar subluxation" OR "subaxilar subluxations" OR "subaxial subluxation" OR "subaxial subluxations" OR "AAI" OR "atlanto axial impaction" OR "atlantoaxial impaction" OR "AAS" OR "atlantoaxial subluxation" OR "anterior atlantoaxial subluxation" OR "anterior atlanto axial subluxation" OR "anterior atlantoaxial subluxations" OR "anterior atlanto axial subluxations" OR "VT" OR "vertical translocation" OR "vertical translation" OR "vertical translocations" OR "vertical translations") AND ts=("DAS28" OR "DAS28" OR "DAS 28" OR "das28 score" OR "das28 scored" OR

"das28 scores" OR "das28 value" OR "das28 values" OR "das 28 score" OR "das 28 scores" OR "das 28 values" OR "disease activity score" OR "disease activity score 28" OR "disease activity" OR ("Disease activity" AND "Scoring System") OR "disease activities" OR "disease active" OR "diseases activities" OR "diseases activity" OR "illness activity" OR "Severity of Illness Index") AND ts=("Time" OR "time" OR time* OR timing* OR "long term" OR "longterm" OR "short term" OR "shortterm" OR followup* OR "follow up" OR "Follow Up"))

Cochrane

http://www.cochranelibrary.com/

(("Rheumatoid Arthritis" OR "rheumatoid arthritis" OR "rheumatoid" OR "rheumatic" OR "arthritis" OR arthriti* OR "RA") AND ("Cervical Vertebra" OR cervical* OR "SAS" OR "subaxilar subluxation" OR "subaxilar subluxations" OR "subaxial subluxation" OR "subaxial subluxations" OR "AAI" OR "atlanto axial impaction" OR "atlantoaxial impaction" OR "AAS" OR "atlantoaxial subluxation" OR "anterior atlantoaxial subluxation" OR "anterior atlanto axial subluxation" OR "anterior atlantoaxial subluxations" OR "anterior atlanto axial subluxations" OR "VT" OR "vertical translocation" OR "vertical translation" OR "vertical translocations" OR "vertical translations") AND ("DAS28" OR "DAS28" OR "DAS 28" OR "das28 score" OR "das28 scored" OR "das28 scores" OR "das28 value" OR "das28 values" OR "das 28 score" OR "das 28 scores" OR "das 28 values" OR "disease activity score" OR "disease activity score 28" OR "disease activity" OR ("Disease activity" AND "Scoring System") OR "disease activities" OR "disease active" OR "diseases activities" OR "diseases activity" OR "illness activity" OR "Severity of Illness Index") AND ("Time" OR "time" OR time* OR timing* OR "long term" OR "longterm" OR "short term" OR "shortterm" OR followup* OR "follow up" OR "Follow Up")):ti,ab,kw

CENTRAL

https://archie.cochrane.org/index.jsp?redirectTo=http://crso.cochrane.org/login.php&key=58c18a4283f80

schoones/

Emcare http://ovidsp.ovid.com/ovidweb.cgi?T=JS&NEWS=n&CSC=Y&PAGE=main&D=emcr

PeDRO http://www.pedro.org.au/

PsycINFO http://search.ebscohost.com/login.aspx?authtype=ip,uid&profile=lumc&defaultdb=psyh

ERIC

Via Ebsco: http://databases.library.leiden.edu/?bibid=990024848320302711&redirect=true

Via OVID: http://ovidsp.tx.ovid.com/sp-3.21.0a/ovidweb.cgi?&S=FKFBFPLFBNDDFEMKNCIKKBGCMKJJAA00&New+Database=Single%7c4

Academic Search Premier [fulltextzoeken]
http://search.ebscohost.com/login.aspx?authtype=ip,uid&profile=lumc&defaultdb=aph

Social Services Abstracts

https://search.proquest.com/socialservices?accountid=12045

http://databases.library.leiden.edu/?bibid=990027333030302711&redirect=true

Sociological Abstracts

https://search.proquest.com/socabs/index

http://databases.library.leiden.edu/?bibid=990024621960302711&redirect=true

WHO International Clinical Trials Registry Platform

http://apps.who.int/trialsearch/

ClinicalTrials.gov

http://clinicaltrials.gov/

+ beta-site: https://clinicaltrials.gov/beta/

European Union Clinical Trials Register

https://www.clinicaltrialsregister.eu/ctr-search/search

Current Controlled Trials

http://www.controlled-trials.com/

Multi-register

http://www.controlled-trials.com/mrct/

Open Trials

http://explorer.opentrials.net/search?q

Prospero

http://www.crd.york.ac.uk/prospero/

Converis

https://converis.lumc.nl/converis/secure/client/login

Journal databases [fulltextzoeken]

A. ScienceDirect

http://www.sciencedirect.com/science?_ob=MiamiSearchURL&_method=requestForm&_temp=all_boolSearch.tmpl&_acct=C000026638&_version=1&_urlVersion=1&_userid=530453&md5=d44bd9fa9076bb9b258a588b309be1e3
